# Diastolic Cardiac Function Improvement by Liraglutide Is Mainly Body Weight Reduction Dependent but Independently Contributes to B-Type Natriuretic Peptide Reduction in Patients with Type 2 Diabetes with Preserved Ejection Fraction

**DOI:** 10.1155/2021/8838026

**Published:** 2021-03-27

**Authors:** Kunimasa Yagi, Teruhiko Imamura, Hayato Tada, Daisuke Chujo, Jianhui Liu, Yuuki Shima, Azusa Ohbatake, Yukiko Miyamoto, Satoko Okazaki, Naoko Ito, Kaoru Nakano, Masataka Shikata, Asako Enkaku, Akiko Takikawa, Hisae Honoki, Shiho Fujisaka, Hideki Origasa, Kazuyuki Tobe

**Affiliations:** ^1^1st Department of Internal Medicine, University of Toyama, 2630 Sugitani, Toyama 934-0194, Japan; ^2^2nd Department of Internal Medicine, Kanazawa University Graduate School of Medical Science, 13-1 Takaramachi, Kanazawa 920-0934, Japan; ^3^2nd Department of Internal Medicine, University of Toyama, 2630 Sugitani, Toyama 934-0194, Japan; ^4^Biostatistics and Clinical Epidemiology, University of Toyama Graduate School of Medicine and Pharmaceutical Sciences, 2630 Sugitani, Toyama 934-0194, Japan

## Abstract

**Objectives:**

A single-arm prospective study was conducted among Japanese patients with type 2 diabetes having preserved ejection fraction. The aim was to investigate (1) whether liraglutide therapy could improve B-type natriuretic peptide (BNP) levels and diastolic cardiac function assessed by the *E*-wave to *E*′ ratio (*E*/*E*′) using transthoracic echocardiography (TTE), and (2) whether *E*/*E*′ contributed to BNP improvement independent of bodyweight reduction (UMIN000005565).

**Methods:**

Patients with type 2 diabetes and left ventricular ejection fraction (LVEF) ≥ 40% without heart failure symptoms were enrolled, and daily injection with liraglutide (0.9 mg) was introduced. Cardiac functions were assessed by TTE before and after 26 weeks of liraglutide treatment. Diastolic cardiac function was defined as septal *E*/*E*′ ≥ 13.0.

**Results:**

Thirty-one patients were analyzed. BNP and *E*/*E*′ improved, with BNP levels declining from 36.8 ± 30.5 pg/mL to 26.3 ± 25.9 pg/mL (*p* = 0.0014) and *E*/*E*′ dropping from 12.7 ± 4.7 to 11.0 ± 3.3 (*p* = 0.0376). The LVEF showed no significant changes. *E*/*E*′ improved only in patients with *E*/*E*′ ≥ 13.0. Favorable changes in *E*/*E*′ were canceled when adjusted for body mass index (BMI). Multivariate linear regression analysis revealed that the left ventricular diastolic diameter and ∆*E*/*E*′/∆BMI contributed to ∆BNP/baseline BNP (*p* = 0.0075, *R*^2^ = 0.49264).

**Conclusions:**

Liraglutide had favorable effects on BNP and *E*/*E*′ but not on LVEF. *E*/*E*′ improvement was only seen in patients with diastolic cardiac function. Body weight reduction affected the change of *E*/*E*′. The BMI-adjusted *E*/*E*′ significantly contributed to the relative change of BNP. GLP-1 analog treatment could be considered a therapeutic option against diabetic diastolic cardiac dysfunction regardless of body weight. This trial is registered with the University Hospital Medical Information Network in Japan, with clinical trial registration number: UMIN000005565.

## 1. Introduction

Heart failure is the leading cause of diabetes-related morbidity and mortality [1]. In patients with diabetes, heart failure with preserved ejection fraction (HFpEF) plays a central role in the occurrence and progression of heart failure [2]. HFpEF is derived from diastolic cardiac dysfunction and is considered a clinical manifestation of the microvascular disease associated with type 2 diabetes [3]. With the clinical significance of antidiabetic agents over the prevention of microvascular diseases [4], pharmacological intervention against hyperglycemia might be a favorable strategy for improving the prognosis of HFpEF with type 2 diabetes. However, some antidiabetic medications can actually increase the risk of heart failure [5, 6]. This finding emphasizes the need for therapies that can address the risks of heart failure in patients with diabetes.

One potential class of medications that may be of value is the glucagon-like peptide 1 (GLP-1) analogs, shown in prospective studies to suppress cardiovascular death from causes including heart failure [7]. The American Diabetes Association (ADA) and the European Association for the Study of Diabetes (EASD) consensus guidelines [8] have also recommended GLP-1 analogs as a second-line medication for glycemic management of type 2 diabetic patients with heart failure. Currently, the European Society of Cardiology (ESC) and EASD guidelines have endorsed GLP-1 analogs and sodium glucose transporter 2 (SGLT2) inhibitors as the first-line therapies, rather than metformin, for patients with diabetes having atherosclerotic cardiovascular disease (ASCVD) or a high risk of cardiovascular events [9].

One GLP-1 analog, liraglutide, was shown to suppress cardiovascular death with a hazard ratio of 0.78 in the LEADER study [10]. Liraglutide also reduced heart failure hospitalization as the secondary outcome of the LEADER study [10] and a health record study [11]. In patients with noncomplicated diabetes, liraglutide improved diastolic cardiac function in retrospective studies [12–14]. Two prospective studies examining acute coronary syndrome [15, 16] and one on end-stage renal disease dependent on peritoneal dialysis [17] have also shown a reduction in the left ventricular mass index at six months following liraglutide introduction. Four randomized liraglutide studies on patients with diabetes showed an improvement in diastolic function. These studies included the LIVE study that assessed 212 participants (106 treated with liraglutide and 106 treated with placebo) for 24 weeks with transthoracic echocardiography (TTE) [18], a study on 32 overweight participants treated with exercise and medication (16 treated with liraglutide and 16 with a placebo) for 16 weeks and assessed with TTE [19], another that assessed 60 participants (30 treated with liraglutide and 30 with metformin) for six months with TTE [20], and a study on 49 participants (23 treated with liraglutide and 26 with a placebo for 26 weeks) that assessed cardiac function with magnetic resonance imaging (MRI) [21].

The favorable effects of liraglutide on diastolic function appear to be well established at present. By contrast, none of the medications typically used in the field of cardiovascular medicine, including diuretics [22], digoxin [23], beta-blockers [24], angiotensin-converting enzyme inhibitor [25], angiotensin II receptor blocker [26], and aldosterone antagonist [27, 28], have shown the capability of altering the prognosis of subjects with diastolic cardiac dysfunction. In the present study, we have focused on body weight reduction by liraglutide, since body weight reduction induces favorable changes in hemodynamics and reduces cardiac preload and afterload, thereby improving diastolic cardiac function [29, 30].

We hypothesized that liraglutide would improve diastolic cardiac function mainly through body weight reduction. No previous clinical studies on GLP-1 analogs have investigated in detail the echocardiographic parameters associated with body weight reduction. Therefore, the primary aim of this study was to examine the contribution of body weight reduction to previously reported findings of heart failure risk reduction by liraglutide. The second aim was to confirm the previously reported findings of the improvement of B-type natriuretic peptide (BNP) levels and of diastolic function assessed by TTE in patients with diastolic dysfunction, and to evaluate the contribution of the diastolic function to BNP.

## 2. Methods

### 2.1. Ethical Consideration

This single-center study was registered beforehand (trial registration number: UMIN000005565). The primary outcome was the changes in serum BNP levels, and the secondary outcomes were the TTE parameters, blood pressure, body weight, and laboratory data following liraglutide treatment for 26 weeks.

The local ethics committee approved the study (IRB 2014124 (1743)). All procedures followed the ethical standards of the Helsinki Declaration. Informed consent was obtained from all patients prior to the study. The patients were recruited with web notification and were informed that they could opt out at any time.

### 2.2. Study Patients

The inclusion criteria were as follows: (1) type 2 diabetes, aged >30 years old, and treated with insulin for more than five years; (2) no use of incretin-based therapy at baseline; (3) tolerable to daily injection of liraglutide; (4) preserved left ventricular ejection fraction (LVEF ≥ 40%); (5) no symptomatic heart failure; (6) no history of heart failure admission; (7) no coronary intervention within three years; and (8) no critical primary heart disease, including ventricular arrhythmias, persistent atrial fibrillation, complete atrioventricular block, cardiomyopathy, and valvular disease. The diagnoses of type 2 diabetes were based on the ADA diagnostic criteria.

The exclusion criteria were as follows: (1) endocrine disorders, including type-1 diabetes; (2) refractory malignant tumors; (3) dependency on hemodialysis; and (4) severe hepatic dysfunction (Child − Pugh Score ≥ 10).

All the patients were administered liraglutide injections at the maximum dose of 0.9 mg per day following the protocol provided by Novo Nordisk, Japan (the maximal liraglutide dosage permitted was 0.9 mg in Japan during the study period). The diabetologists adjusted the participants' insulin doses to achieve fair HbA1c levels of <7.0% and to avoid hypoglycemia (i.e., plasma glucose levels below 70 mg/dL). The pharmacists educated the participants on the use of liraglutide during the admission period. All patients continued their daily liraglutide injections throughout the study period. The patients' records ensured adherence and persistence.

### 2.3. Data Collection

All patients had undergone repeated TTE at a clinically stable condition. The TTE recordings and measurements were performed following the American Society of Echocardiography guidelines, using a standard imaging transducer (EPIQ; Philips, Inc., Netherland) with a linear probe frequency of 5 MHz.

The initial TTE was performed within three months before the liraglutide introduction. The second TTE was conducted after 26 ± 4 weeks following the liraglutide introduction. Two cardiologists independently evaluated the data. Standard echocardiographic parameters were obtained from M-mode imaging. The ratio of the peak early diastolic (*E*) and the peak atrial systolic (*A*) transmitral flow velocities (*E*/*A*) and deceleration time were calculated. The *E*-wave to *E*′ ratio (*E*/*E*′) was calculated using tissue Doppler imaging. Routine echocardiographic evaluations included the left ventricular diastolic diameter (LVDd), the left ventricular systolic diameter (LVEF), the left atrial dimension, and *E*/*E*′.

Body weight was measured with a precisely controlled weight scale (Tanita, Japan). Fasting glucose, HbA1c, serum creatinine, lipid levels, urine albumin-to-creatinine ratio, and BNP were measured at the same time as the TTE evaluations, using standard laboratory procedures. The plasma levels of BNP were measured using a commercially available kit (Shionoria BNP Kit, Shionogi Pharmaceutical, Osaka, Japan). The use of medications was collected from medical records.

#### 2.3.1. Definition of Diastolic Cardiac Dysfunction

We defined diastolic cardiac dysfunction as satisfying both increased *E*/*E*′ (septal *E*/*E*′ ≥ 13.0) and preserved LVEF (LVEF ≥ 40%) [31, 32].

### 2.4. Statistical Analysis

The number of study patients needed was calculated to detect BNP's significant changes based on our preliminary examination and the advice from our former colleagues. They later published a liraglutide study on diastolic cardiac dysfunction [15]. With the expected mean difference of BNP as ten and its standard deviation as twenty, the effective size (ES) would be 0.5 (ES = 10/20). Lehr's formula, with an estimation for a power of 80% and an *α* of 0.05, would then determine the requisite number of the patients as 32.

Continuous variables are expressed as means ± standard deviations, and categorical variables are expressed as numbers and percentages. Differences in the baseline characteristics were evaluated using Student's *t*-test for parametric data. Categorical variables were compared using the Chi-square test or Fisher's exact test. The paired data were compared using the paired *t*-test for continuous data and the Wilcoxon signed-rank test for categorical data. The association was evaluated by univariate analysis. Multivariate linear regression analysis was used to assess independent contributors. The relative change was evaluated by adjusting for the baseline value. The correlated parameters were adjusted by division. A *p* value < 0.05 was considered statistically significant, and all tests were two-tailed. Statistical analyses were performed with JMP Pro ver. 15.1.

## 3. Results

### 3.1. Baseline Characteristics

Participants were enrolled between May 2011 and December 2013, with the last patient visit occurring in June 2014. A total of 42 patients were screened. Two patients withdrew because of recurrent gastrointestinal symptoms. Four missed TTE at 26 ± 4 weeks from the liraglutide introduction and were excluded. Five others were excluded due to newly recognized paroxysmal atrial fibrillation (Paf). Ultimately, 31 patients were analyzed in this study.

The baseline characteristics of the 31 patients (65 ± 12 years old, 21 males) are shown in Table 1. The use of insulin started in all 31 patients at 28 ± 21 IU/day at baseline and changed in 7 patients at 16 ± 12 IU/day at 26 weeks. The medications were not changed for any patient during the study period. Gender, duration of diabetes, BMI, blood pressure, use of medications, lipid levels, diabetic complications, and glycemic control were comparable in both groups with and without diastolic dysfunction, but the patients with diastolic cardiac dysfunction were older.

### 3.2. Comparisons of Clinical Parameters

**Figure 1:**
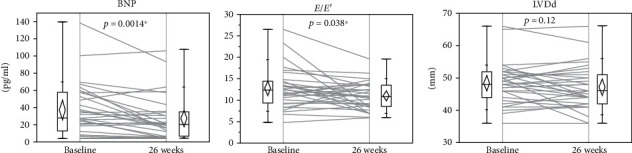
Comparison of clinical parameters at baseline and 26 weeks after liraglutide introduction (*N* = 31). Changes in (a) BNP, (b) *E*/*E*′, and (c) LVDd in study subjects. BNP: B-type natriuretic peptide; *E*/*E*′: *E* wave to *E*′ ratio on tissue Doppler echocardiography; LVDd: left ventricular diastolic diameter.

Comparisons of clinical settings at baseline and at 26 weeks are shown in Table 2. HbA1c, BMI, and diastolic blood pressure were reduced. HbA1c improved from 7.5 ± 1.2% (57 ± 12 mmol/mol) to 6.9 ± 0.9% (50 ± 9 mmol/mol) (*p* = 0.0003), BMI dropped from 26.2 ± 4.9 kg/m^2^ to 25.0 ± 4.8 kg/m^2^ (*p* < 0.0001), BNP levels improved from 36.8 ± 30.5 pg/mL to 26.3 ± 25.9 pg/mL (*p* = 0.0014; Figure 1(a)), and *E*/*E*′ improved from 12.7 ± 4.7 to 11.0 ± 3.3 (*p* = 0.0376; Figure 1(b)). The other TTE indices, including LVEF and LVDd (Figure 1(c)), showed no significant changes.

### 3.3. Differences in TTE Parameter Changes between the Patients with and without Elevated *E*/*E*′

**Figure 2:**
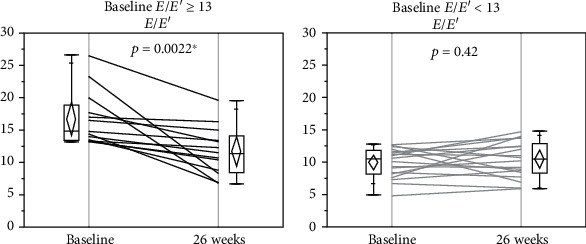
Comparison of *E*/*E*′ at baseline and 26 weeks after liraglutide introduction in patients with *E*/*E*′ ≥ 13 (*N* = 13) and those with *E*/*E*′ < 13 (*N* = 18). Changes in *E*/*E*′ (a) in patients with *E*/*E*′ ≥ 13 and (b) in patients with *E*/*E*′ < 13. *E*/*E*′: *E* wave to *E*′ ratio on tissue Doppler echocardiography.

**Figure 3:**
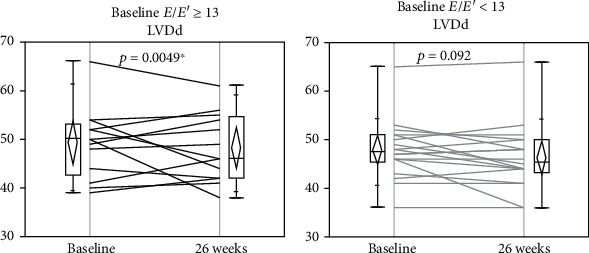
Comparison of LVDd at baseline and 26 weeks after liraglutide introduction in patients with *E*/*E*′ ≥ 13 (*N* = 13) and those with *E*/*E*′ < 13 (*N* = 18). Changes in LVDd (a) in patients with *E*/*E*′ ≥ 13 and (b) in patients with *E*/*E*′ < 13. *E*/*E*′: *E* wave to *E*′ ratio on tissue Doppler echocardiography; LVDd: left ventricular diastolic diameter.

Improvements in the *E*/*E*′ and LVDd were observed in separate subanalyses in the patients with *E*/*E*′ ≥ 13 (Figures 2(a) and 3(a)) but not in those with *E*/*E*′ < 13 (Figures 2(b) and 3(b)). In other words, the favorable changes induced by liraglutide were observed only in the patients with diastolic cardiac dysfunction.

### 3.4. The Effect of Body Weight Reduction on TTE Parameter Changes

**Figure 4:**
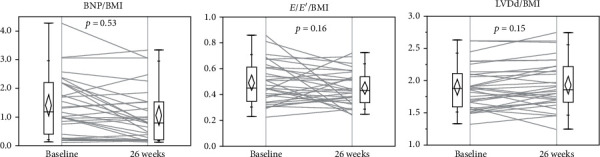
Changes in clinical parameters adjusted by BMI at baseline and 26 weeks after liraglutide introduction. Changes in (a) BNP, (b) *E*/*E*′, and (c) LVDd adjusted by BMI in the subjects. BNP: B-type natriuretic peptide; BMI: body mass index; *E*/*E*′: *E* wave to *E*′ ratio on tissue Doppler echocardiography; LVDd: left ventricular diastolic diameter.

BNP, *E*/*E*′, and LVDd showed no significant changes when divided by the BMI (Figures 4(a)–4(c)). The favorable changes in BNP, *E*/*E*′, and LVDd were canceled when adjusted for the changes in body weight reduction.

### 3.5. Significant Contribution of ∆LVDd, ∆*E*/*E*′, and ∆BMI to ∆BNP

**Figure 5:**
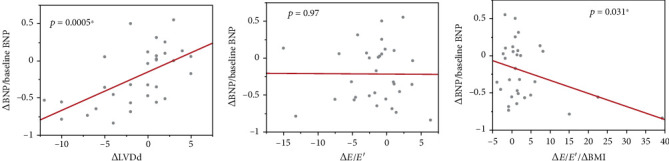
Relationship between relative changes in BNP and changes in LVDd, *E*/*E*′, and *E*/*E*′ adjusted by BMI. Relationship between relative changes in BNP and the changes in (a) LVDd, (b) *E*/*E*′, and (c) *E*/*E*′ divided by BMI in study subjects from baseline to 26 weeks after liraglutide introduction. BNP: B-type natriuretic peptide; BMI: body mass index; *E*/*E*′: *E* wave to *E*′ ratio on tissue Doppler echocardiography; LVDd: left ventricular diastolic diameter.

Correlation analysis showed that ∆BNP/baseline BNP correlated significantly with ∆LVDd (Figure 5(a)) and ∆*E*/*E*′/∆ BMI (Figure 5(c)), but not with ∆*E*/*E*′ (Figure 5(b)). Multivariate linear regression analysis showed the independent contributions of ∆LVDd and ∆*E*/*E*′/∆BMI to ∆BNP/baseline BNP, while ∆BNP/baseline BNP was an objective variable and independent variable of ∆LVDd, ∆*E*/*E*′/∆BMI, ∆EF, ∆HR, ∆dBP, and ∆HbA1c (*p* = 0.0075, *R*^2^ = 0.49264; Table 3). ∆LVDd and ∆*E*/*E*′ adjusted with ∆BMI significantly contributed to the relative change in BNP. The *R*^2^ value of 0.49 showed goodness of fit for the multivariate linear regression model.

## 4. Discussion

This prospective single-arm study showed that a 26-week treatment of liraglutide improved diastolic cardiac function in patients with type 2 diabetes with preserved ejection fraction. The main findings of this study were as follows: (1) liraglutide injection at 0.9 mg per day improved BNP and *E*/*E*′ but did not change LVEF; (2) improvement of *E*/*E*′ occurred only in patients with diastolic cardiac dysfunction; (3) improvement of *E*/*E*′ was canceled when adjusted for bodyweight reduction; and (4) correlation and multivariate linear regression analyses showed that changes in BMI-adjusted *E*/*E*′ and LVDd significantly contributed to the relative change in BNP.

Liraglutide improved diastolic cardiac function mainly in a bodyweight reduction-dependent manner, but it significantly contributed to BNP reduction in a bodyweight reduction-independent manner in patients with type 2 diabetes with preserved ejection fraction. The results were compatible with previous papers that showed an improved diastolic cardiac function and no improvement in systolic cardiac function with liraglutide administration [18, 33]. Additionally, an earlier paper showed that diastolic dysfunction is more prominently related to NIDDM (current type 2 diabetes) than IDDM (current type 1 diabetes) [34]. Insulin resistance due to increased body weight plays a central role in type 2 diabetes [35]. Taken together, these lines of findings and considerations supported the bodyweight dependency of diastolic dysfunction in the subjects with diabetes and its improvement by bodyweight reduction.

This study is the first to examine the contribution of body weight reduction to the change in diastolic function by GLP-1 analogs. The improvement in *E*/*E*′ was canceled when adjusted for body weight reduction; therefore, the previous papers would have overestimated the extent of diastolic cardiac function improvement by GLP-1 analogs because the contribution of body weight reduction was not factored into the estimates. Furthermore, weight reduction should receive more attention as a therapeutic option for diastolic cardiac dysfunction in type 2 diabetes. Interestingly, the extent of *E*/*E*′ improvement in the current study was very similar to that reported in the LIVE study [18], although the liraglutide doses differed (0.9 mg per day in the present study vs. 1.8 mg per day in the LIVE study). We supposed that the effect of liraglutide on *E*/*E*′ could reach a plateau in different dose levels between Asians and Europeans, mainly due to the tolerability against the medication [36].

The improvement in LVDd was difficult to interpret because BNP and LVDd interact, so an improvement in LVDd would result in an improvement in BNP, and vice versa. We could view the improvement in both BNP and LVDd as reflecting the reduction in LV filling pressure. LVDd did not show any significant change following the liraglutide introduction as a whole. LVDd in each patient might have paralleled with the change of BNP levels independently from that of *E*/*E*′.

One safety concern that should be mentioned is Paf. Five patients were newly identified to have Paf following the introduction of liraglutide. We supposed that some cases had unidentified Paf before the study, and this became obvious after liraglutide introduction. GLP-1 analogs can increase the heart rate through sympathetic nervous stimulation [37], although no significant elevation of heart rate was observed in the present study. Asians have a much higher overall disease burden of Paf because of the proportionally larger aged population [38]. Further large-scale examination of Paf related to liraglutide should be conducted.

The GLP-1 analog liraglutide has a beneficial effect on the cardiovascular system [39], since GLP-1 receptors are expressed in endothelial and smooth muscle cells of systemic microvasculature [40] and cardiac ventricles [41]. GLP-1 analogs can improve diastolic function through suppression of cardiac fibrosis [42] and relaxation of vascular smooth muscle and the myocardium by nitric oxide generation [43]. The latter hemodynamic mechanism would have made the main contribution to our study because of the short-term improvement of BNP and *E*/*E*′ and the contribution of cardiac morphological changes reflected by LVDd. The vascular and myocardial relaxation would reduce end-diastolic filling pressure, thereby improving diastolic cardiac function. A recent report has shown that the vasodilatory actions of GLP-1 analogs are preserved in the myocardial microvasculature but not in obese subjects with vascular insulin resistance [44]. Body weight reduction might therefore also have improved the sensitivity of the vascular cells to liraglutide.

Hyperglycemia could affect the prognosis of diabetic patients with diastolic cardiac dysfunction [45]. Patients with type 2 diabetes belong to a high-risk group for heart failure; therefore, proactive examination of the physical findings and noninvasive imaging evaluation is required, as well as consideration of the indications for GLP-1 analogs and SGLT2 inhibitors in light of the heart failure risk exacerbation in this particular subgroup of patients. The indications for GLP-1 analogs for patients with type 2 diabetes should be widened from glycemic control to include the pleiotropic effects on cardiac function and cardiac morphology.

Our results show that the primary candidates for the use of GLP-1 analogs would be the subjects with elevated BNP and *E*/*E*′. Considering the negative consequences of the prospective randomized studies, the FIGHT study, and the LIVE study, patients with reduced EF would not be good candidates for GLP-1 analogs. These medications could be one of the therapeutic options against diastolic cardiac dysfunction, although the extent of improvement might be clinically small. We believe that GLP-1 analogs could be a therapeutic option for HFpEF in patients with type 2 diabetes, but a substantial-scale clinical study would be needed to obtain positive results.

This study has several limitations. One limitation of the present study was its relatively small sample size cohort. However, too large a sample size would be unsuitable for evaluating clinically significant differences in diastolic function improvement. The cancelation of the statistical significance of *E*/*E*′ by body weight reduction adjustment suggested an appropriate sample size calculation in the present study. Another limitation was the absence of a control group and the high heterogeneity in the participants. Another limitation was the enrollment of asymptomatic patients, so the implication of GLP-1 analogs on symptomatic cohorts remains uninvestigated. The applicability of our findings to patients with symptomatic diastolic cardiac dysfunction requires further investigation.

## 5. Conclusions

Liraglutide improves diastolic cardiac function, mainly in a body weight reduction-dependent manner. However, it contributes to the reduction in heart failure risk in patients with type 2 diabetes with preserved ejection fraction independent of body weight reduction. GLP-1 analog treatment should, therefore, be considered as a therapeutic option against diabetic diastolic cardiac dysfunction regardless of a patient's body weight.

Further randomized controlled trials with larger sample sizes that consider HFpEF are needed to establish this pleiotropic effect of GLP-1 analogs. The results would be meaningful as they could widen the therapeutic options for treatment of high heart failure risk in patients with diabetes.

## Figures and Tables

**Table 1:** Patient baseline characteristics.

	Total (*N* = 31)	With DD (*N* = 13)	Without DD (*N* = 18)	*p* value
*Demographics*				
Male gender (%)	21 (68%)	9 (69%)	12 (67%)	0.88
Age (years)	65 ± 12	70 ± 8	62 ± 13	0.036^∗^
Diabetes duration (years)	19 ± 12	20 ± 11	18 ± 12	0.59
Body mass index (kg/m^2^)	26.2 ± 4.9	26.5 ± 4.7	26.0 ± 5.1	0.80
Systolic blood pressure (mmHg)	123 ± 16	124 ± 14	123 ± 18	0.90
Diastolic blood pressure (mmHg)	70 ± 10	68 ± 12	71 ± 9	0.44
Heart rate (bpm)	70 ± 12	67 ± 7	73 ± 13	0.12
B-type natriuretic peptide (pg/mL)	36.8 ± 30.5	40.0 ± 28.3	34.4 ± 32.5	0.62
Blood glucose (mg/dL)	155 ± 48	172 ± 51	143 ± 43	0.099
HbA1c (%)	7.5 ± 1.2	7.3 ± 0.8	7.6 ± 1.5	0.51
Total cholesterol (mg/dL)	170 ± 47	159 ± 34	177 ± 54	0.30
LDL cholesterol (mg/dL)	96 ± 31	92 ± 33	99 ± 30	0.53
HDL cholesterol (mg/dL)	48 ± 15	46 ± 16	49 ± 15	0.56
Triglyceride (mg/dL)	127 ± 104	105 ± 43	142 ± 130	0.32
Creatinine (mg/dL)	0.9 ± 0.4	0.9 ± 0.4	0.9 ± 0.4	0.92
*Diabetic complications*				
Diabetic retinopathy	16 (52%)	8 (62%)	8 (44%)	0.35
Albumin-creatinine rate (mg/g Cr)	292 ± 636	372 ± 671	231 ± 621	0.56
Ischemic heart disease	18 (58%)	9 (69%)	9 (50%)	0.28
Old myocardial infarction	6 (19%)	4 (31%)	2 (11%)	0.17
Cerebrovascular disease	5 (16%)	2 (15%)	3 (17%)	0.92
*Medications*				
Angiotensin II receptor blockers	23 (74%)	11 (85%)	12 (67%)	0.26
Beta-blockers	9 (29%)	4 (31%)	5 (28%)	0.86
Calcium channel blockers	19 (61%)	9 (69%)	10 (56%)	0.44
Loop diuretics	11 (35%)	5 (38%)	6 (33%)	0.77
Statins	22 (71%)	8 (62%)	14 (78%)	0.33
Antiplatelet agents	19 (61%)	8 (62%)	11 (61%)	0.98

Data are means ± standard deviations or *n* (%) unless otherwise specified. DD: diastolic cardiac dysfunction at baseline; HDL: high-density lipoprotein; LDL: low-density lipoprotein; Cr: creatinine; TTE: transthoracic echocardiography; NA: not applicable. ^∗^*p* < 0.05.

**Table 2:** Comparison of clinical parameters at baseline and after 26 weeks of liraglutide treatment.

	Baseline	26 week	*p* value
HbA1c (%)	7.5 ± 1.2	6.9 ± 0.9	0.0003^∗^
Creatinine (mg/dL)	0.9 ± 0.4	0.9 ± 0.4	0.55
Systolic blood pressure (mmHg)	123 ± 16	124 ± 12	0.88
Diastolic blood pressure (mmHg)	70 ± 10	64 ± 9	0.013^∗^
Heart rate (bpm)	70 ± 12	72 ± 9	0.43
Body mass index (kg/m^2^)	26.2 ± 4.9	25.0 ± 4.8	<0.0001^∗^
B-type natriuretic peptide (pg/mL)	36.8 ± 30.5	26.3 ± 25.9	0.0014^∗^
Septal *E*/*E*′	12.7 ± 4.7	11.0 ± 3.3	0.038^∗^
Proportion of diastolic cardiac dysfunction	13 (42%)	9 (29%)	0.33
E/A ratio	0.86 ± 0.27	0.83 ± 0.29	0.46
Deceleration time (msec)	239 ± 59	243 ± 60	0.72
Left atrial diameter (mm)	40.0 ± 8.1	40.4 ± 8.1	0.48
Left ventricular diastolic diameter (mm)	48.4 ± 6.5	47.1 ± 6.8	0.12
Left ventricular ejection fraction (%)	67.5 ± 10.0	65.7 ± 11.1	0.43

Data are means ± standard deviations or *n* (%) unless otherwise specified. *E*/*E*′: *E* wave to *E*′ ratio on tissue Doppler echocardiography; *E*/*A*: ratio of transmitral *E* wave velocity to transmitral *A* wave ratio on transmitral flow image. ^∗^*p* < 0.05.

**Table 3:** Multivariate linear regression to relative changes in B-type natriuretic peptide.

Variable	Coefficient	Standard error	*p* value
∆Left ventricular diastolic diameter	3.146	0.014	0.0007^∗^
∆*E*/*E*′/∆BMI	1.570	0.007	0.027^∗^
∆Left ventricular ejection fraction	0.444	0.007	0.36
∆Heart rate	0.276	0.007	0.53
∆Diastolic blood pressure	0.106	0.005	0.78
∆HbA1c	0.056	0.072	0.88

*E*/*E*′: *E* wave to *E*′ ratio on tissue Doppler echocardiography; BMI: body mass index. ^∗^*p* < 0.05.

## Data Availability

The data used to support the findings of this study are available from the corresponding author upon request.

## Abbreviations

BMI: Body mass index
BNP: B-type natriuretic peptide
dBP: Diastolic blood pressure
*E*/*E*′: *E*-wave to *E*′ ratio on tissue Doppler echocardiography
HFpEF: Heart failure with preserved ejection fraction
LVDd: Left ventricular diastolic diameter
LVEF: Left ventricular ejection fraction.
